# Virulence Factor Genes and Cytotoxicity of *Streptococcus agalactiae* Isolated from Bovine Mastitis in Poland

**DOI:** 10.1128/spectrum.02224-21

**Published:** 2022-05-24

**Authors:** Ewa Zastempowska, Magdalena Twarużek, Jan Grajewski, Henryka Lassa

**Affiliations:** a Department of Physiology and Toxicology, Faculty of Biological Sciences, Kazimierz Wielki University, Bydgoszcz, Poland; b University of Bydgoszcz, Bydgoszcz, Poland; University of Adelaide

**Keywords:** virulence genes, *in vitro* cytotoxicity, MTT test, LDH test

## Abstract

Streptococcus agalactiae can produce a wide variety of virulence factors, including toxins and proteins which facilitate adhesion to and colonization and invasion of the host cells. There are few reports on the characteristics of field isolates from bovine mastitis in Poland. Thus, the aim of this study was to determine the occurrence of types of hemolysis on blood agar, virulence factor genes, and cytotoxicity of S. agalactiae isolates derived from cows with mastitis across Poland. The study included 68 isolates. Virulence genes were tested using standard PCR, and cytotoxicity was determined using methylthiazol tetrazolium (MTT) and lactate dehydrogenase (LDH) tests. Among the tested isolates, 89.7% were β-hemolytic, 8.8% γ-hemolytic, and 1.5% alpha-hemolytic. The only genes detected in all isolates were the *cfb*, *cspA*, *hylB*, and *sip* genes. Cytotoxicity assessment based on the LDH test revealed that isolates were cytotoxic only to Vero cells. However, according to the results obtained from the MTT test, more than half of the isolates exhibited low cytotoxicity to both SK and Vero cells, whereas the other isolates showed moderate or no cytotoxicity to both cell lines. Our research confirms the prevalence of various virulence genes in S. agalactiae isolated from Polish dairy herds, which have previously been found in isolates recovered from human and animal infections. For the first time, the presence of *bac*- and *scpB*-positive isolates of S. agalactiae was determined in Polish dairy cattle, and the cytotoxicity of bovine isolates was assessed.

**IMPORTANCE** We believe that this manuscript is one of the few reports on the characteristics of field S. agalactiae isolates derived from cases of bovine mastitis in cows in Poland in terms of the occurrence of virulence genes and cytotoxicity. For the first time, the presence of *bac*- and *scpB*-positive isolates of S. agalactiae was determined in Polish dairy cattle, and the cytotoxicity of bovine isolates was assessed.

## INTRODUCTION

Streptococcus agalactiae (group B Streptococcus; GBS) is the main cause of infections in human neonates and young infants (1). It is also an important opportunistic pathogen which colonizes the gastrointestinal and genitourinary tracts, throat, and skin of healthy adults (2). In cattle, Streptococcus agalactiae is a major etiological agent of clinical and subclinical mastitis (3), a disease of economic importance (4). Over the past few decades, the incidence of udder inflammation caused by S. agalactiae has decreased due to the introduction of mastitis control programs in developed dairy countries. However, in recent years, in some European countries such as Denmark and Finland, this species has increasingly been isolated from bovine mastitis (5, 6). Streptococcus agalactiae also poses a threat for many other countries, especially those with an emerging dairy industry, such as Brazil and Colombia (3, 7). Until recently, S. agalactiae has been considered an obligate intramammary pathogen, strictly contagious in cows (8). In studies conducted over the past few years, S. agalactiae has been isolated from sites other than the bovine udder, such as bovine feces, the throats of calves, the vaginas of lactating cows, and the environment of the animals (8–10).

S. agalactiae may be capable of producing a wide variety of virulence factors, including toxins (β-hemolysin/cytolysin and CAMP factor), proteins facilitating adhesion to and colonization and invasion of the host cells (FbsA fibrinogen-binding protein, αC protein, Rib protein, and HylB hyaluronidase), factors determining resistance to antibacterial peptides (βC protein), and factors which enable avoidance of the host immune response (ability to produce C5a peptidase, CspA serine protease) (11).

GBS β-hemolysin/cytolysin plays a role in tissue damage (cytolytic function) and allows bacteria to enter epithelial and endothelial cells, contributing to overcoming the blood-brain barrier (12, 13). Another S. agalactiae toxin, the CAMP factor, is an extracellular protein which causes the formation of pores in target cells (14).

FbsA and FbsB are proteins which both bind to human fibrinogen, mediate bacterial adhesion to or invasion of host cells, and contribute to escape from the immune system. FbsA promotes adhesion to epithelial and endothelial cells (15), whereas FbsB facilitates host epithelial cell invasion by S. agalactiae (15, 16). S. agalactiae hyaluronidase HylB has been assumed to facilitate the spread of the organism through the tissues of the infected host. It also has a strong influence on the intracellular survival of these bacteria and stimulation of proinflammatory cytokine secretion, and also plays a nutritional role (17). The CspA serine protease is involved in the cleavage of fibrinogen (18) and the degradation of chemokines (19). The surface proteins of S. agalactiae include the Sip, C, and Rib proteins. The C protein consists of two antigens, αC and βC, the first of which, encoded by the *bca* gene, is involved in the penetration of bacteria into host epithelial cells (20). The βC antigen, encoded by the *bac* gene, can bind IgA antibodies and human factor H (21).

The C5a peptidase (serine protease), encoded by the *scpB* gene, is involved in the binding of fibronectin and causes specific inactivation of the C5a component of human complement (22).

So far, no large-scale studies have been conducted in Poland to assess the properties of field S. agalactiae isolates derived from cases of bovine mastitis in cows across the country, which would take into account the presence of virulence genes and cytotoxicity levels of the isolates. In this context, we aimed to determine the occurrence of types of hemolysis and virulence factor genes, and to assess the cytotoxicity of S. agalactiae isolates derived from cows with mastitis across Poland.

## RESULTS

### Type of hemolysis on blood medium.

On agar medium with 5% sheep blood, 61 S. agalactiae isolates (89.7%) produced β-type hemolysis. Six isolates (8.8%) showed the presence of γ-type hemolysis, whereas one isolate was alpha-hemolytic (1.5%).

### Occurrence of virulence genes.

In all tested S. agalactiae isolates, the presence of a gene fragment encoding CspA serine protease, the HylB hyaluronidase gene, and the Sip protein were detected. All isolates were also *cfb*-positive and produced CAMP factor on sheep blood medium. Other virulence genes were not present in all isolates. Most of the isolates were characterized by a fragment of the gene encoding the fibrinogen-binding protein FbsA (92.6%) and the structural gene of β-hemolysin/cytolysin (95.6%). The *cylE* gene was present in the alpha-hemolytic isolate and in all β-hemolytic isolates, whereas it was absent in three of the six γ-hemolytic isolates in the medium supplemented with sheep blood. The genes for C5a peptidase (*scpB*) and Rib protein (*rib*) production, as well as the genes encoding the α (*bca*) and β antigens (*bac*) of the C surface protein, were present in less than half of the S. agalactiae isolates (Table 1).

**TABLE 1:** Occurrence of virulence genes in bovine Streptococcus agalactiae isolates (*n* = 68) from clinical and subclinical mastitis

Virulence gene	Clinical mastitis (*n* = 48), *n* (%)	Subclinical mastitis (*n* = 20), *n* (%)	*P* ^*a*^	Total
*bac*	10 (20.8)	1 (5.0)	0.1062	11/68
*bca*	5 (10.4)	1 (5.0)	0.4730	6/68
*cylE*	45 (93.8)	20 (100)	0.6202	65/68
*fbsA*	45 (93.8)	18 (90.0)	0.5893	63/68
*rib*	5 (10.4)	2 (10.0)	0.9589	7/68
*scpB*	16 (33.3)	8 (40.0)	0.6002	24/68
*cfb*	48 (100)	20 (100)		68/68
*cspA*	48 (100)	20 (100)		68/68
*hylB*	48 (100)	20 (100)		68/68
*sip*	48 (100)	20 (100)		68/68

a Statistical analysis was performed only for virulence factors which were not present in all Streptococcus agalactiae isolates. A *P *value of ≤* *0.05 or ≤0.001 was considered statistically significant.

The presence of virulence genes in isolates derived from clinical and subclinical cases of mastitis was compared. Some genes (*bac*, *bca*, *fbsA*) were encountered more frequently among isolates derived from clinical mastitis than in those from subclinical mastitis. However, the presence of the tested virulence genes had no statistically significant association with the type of mastitis. The relationship between the presence of individual virulence genes and inflammation of a cow’s udder was low (Table S1 in the supplemental material).

Among the tested S. agalactiae isolates, 13 profiles of detected virulence genes were found. The most common genotype was *cfb cspA cylE fbsA hylB sip* (45.6% of isolates) (Table 2).

**TABLE 2:** Virulence gene profiles of Streptococcus agalactiae isolated from cows presenting clinical or subclinical mastitis (68 isolates)

Profile no.	Genes	No. of isolates, *n* (%)
1	*bac bca cfb cspA cylE fbsA hylB scpB sip*	4 (5.9)
2	*bac bca cfb cspA cylE fbsA hylB sip*	1 (1.5)
3	*bac cfb cspA cylE fbsA hylB scpB sip*	3 (4.4)
4	*bac cfb cspA cylE fbsA hylB sip*	3 (4.4)
5	*bca cfb cspA cylE fbsA hylB scpB sip*	1 (1.5)
6	*cfb cspA cylE fbsA hylB rib sip*	5 (7.3)
7	*cfb cspA cylE fbsA hylB scpB sip*	13 (19.1)
8	*cfb cspA cylE fbsA hylB sip*	31 (45.6)
9	*cfb cspA cylE hylB scpB sip*	3 (4.4)
10	*cfb cspA cylE hylB sip*	1 (1.5)
11	*cfb cspA fbsA hylB rib sip*	1 (1.5)
12	*cfb cspA fbsA hylB sip*	1 (1.5)
13	*cfb cspA hylB rib sip*	1 (1.5)

### Cytotoxicity of *S. agalactiae* isolates.

The cytotoxicity assessment based on the amount of lactate dehydrogenase (LDH) released into the medium revealed that S. agalactiae isolates were cytotoxic only to Vero cells. However, according to the results obtained from the methylthiazol tetrazolium (MTT) test, more than half of the isolates exhibited low cytotoxicity to both SK and Vero cells, whereas the other isolates showed moderate or no cytotoxicity to both cell lines. There were no isolates with high cytotoxicity to either examined cell line (Table 3).

**TABLE 3:** Results of cytotoxicity assays which included Streptococcus agalactiae isolates (*n* = 34) derived from bovine clinical and subclinical mastitis

Cytotoxicity assay	Cell line	No. of bacterial isolates, *n* (%)			
		Cytotoxicity^*a*^			
		None	Low	Moderate	High
MTT	SK	1 (2.9)	18 (52.9)	15 (44.1)	0 (0)
	Vero	8 (23.5)	18 (52.9)	8 (23.5)	0 (0)
				
LDH	SK	34 (100)	0 (0)	0 (0)	0 (0)
	Vero	19 (55.9)	15 (44.1)^*b*^		

a Cytotoxicity: low, <50%; moderate, 50 to 85%; high, >85%.

b The value “15 (44.1)” refers to all levels of cytotoxicity.

Statistical analyses were performed to determine the correlation of the presence of virulence genes with cytotoxicity toward the SK and Vero cell lines. The presence of virulence genes was not found to be associated with cytotoxicity toward SK and Vero cells according to the MTT test. However, only the presence of the *scpB* gene had a significant effect on the cytotoxicity of isolates to Vero cells (χ^2^_Yates_ = 6.94; *P* = 0.009). A statistically significant and moderate positive correlation was found between the presence of the *scpB* virulence gene and the cytotoxicity of isolates toward Vero cells (*R_Spr_* = 0.42; *P* = 0.013) (supplemental material, Table S2). No correlation was found between virulence genes and cytotoxicity to Vero cells according to the LDH test. Due to the lack of variability for cytotoxicity, the SK cell line could not be analyzed using the LDH test (supplemental material, Table S3).

## DISCUSSION

In our study, 68 isolates were identified as S. agalactiae using standard microbiological methods, biochemical tests, and standard PCR. The identification methodology of this study was not ideal, yet it is frequently used in many commercial diagnostic laboratories. Matrix-assisted laser desorption ionization–time of flight mass spectrometry (MALDI-TOF MS) and/or whole-genome sequencing would be better (23). In this study, all S. agalactiae isolates derived from cows presenting either clinical or subclinical mastitis were screened for the presence of the genes encoding several known and putative virulence factors which seem to be associated with pathogenicity in these bacteria. One of these virulence factors, β-hemolysin/cytolysin, is responsible for the appearance of a characteristic β-hemolysis zone on blood agar. In our own research, the vast majority of S. agalactiae isolates produced β-type hemolysis on sheep blood (89.7%); fewer isolates were nonhemolytic (forming γ-hemolysis) (8.8%), and alpha-hemolytic isolates were rarely isolated (1.5%). These results contradict the findings of previously conducted studies in which, among S. agalactiae isolated from the milk of cows with clinical or subclinical mastitis, slightly more than half of the isolates were beta-hemolytic (52.9%) (24). In this study, almost all studied isolates were characterized by the presence of the structural gene of β-hemolysin/cytolysin (*cylE*) (95.6% of isolates). The gene was not detected in three of the six nonhemolytic isolates. The remaining three isolates, despite the presence of the *cylE* gene, did not produce β-type hemolysis, which could be due to a lack of expression of the gene. Some studies have demonstrated the presence of the *cylE* gene in all tested isolates from dairy cattle with clinical or subclinical mastitis (25, 26) and clinical forms of the disease (20). On the other hand, some authors have detected the *cylE* gene in lower numbers of S. agalactiae isolates from subclinical (22.2%) (27) and clinical cases of bovine mastitis (78%) (28).

Group B streptococcus can be initially identified based on a positive result of a CAMP test, which is based on the detection of the diffusing CAMP factor produced by the majority (95 to 97%) of S. agalactiae strains (29). Incorrect identification of the etiological agents of bovine mastitis may lead to inappropriate management, highlighting the need to identify microorganisms isolated from milk using rapid and accurate molecular biology techniques. The CAMP factor-encoding gene (*cfb*) is the most frequently used target for PCR assays which seemed to be present in all GBS strains (30, 31); however, a recent study has reported the occurrence of a *cfb*-negative S. agalactiae isolate (31). Our own research also showed the presence of the *cfb* gene and the CAMP protein in all S. agalactiae isolates. The same results have been obtained in other studies on S. agalactiae from bovine clinical mastitis (28); however, some authors reported the presence of the *cfb* gene in 98.8% of isolates from clinical mastitis milk samples (32). Our results and those obtained by other authors suggest that this toxin is highly important in S. agalactiae infections. However, a previous study performed using a murine infection model indicates that it does not play a significant role in systemic GBS virulence (14).

The other virulence genes found in all isolates tested in our study include the *hylB* gene, encoding HylB hyaluronidase, and the *cspA* gene, encoding the CspA serine protease. Previous studies have also shown that the *hylB* gene has been detected in all isolates from milk of cows with clinical or subclinical mastitis (25, 26). The same reports revealed the presence of the *cspA* gene in all (25) or in the vast majority (96%) (26) of isolates from dairy cows with mammary gland inflammation.

Most isolates tested in our study also showed the presence of the *fbsA* gene, encoding a fibrinogen-binding protein FbsA (92.6% of isolates). Previously, the gene was less prevalent (42.4%) among S. agalactiae isolates from mastitis (clinical or subclinical form) in Brazilian dairy herds (3). Interestingly, different results were obtained by Rato et al. (33), who did not detect this gene in any of the tested S. agalactiae isolates from the milk of cows with subclinical mastitis in Portugal, which may result from the different origin of these isolates. Both studies (3, 33) reported that all isolates were characterized by the presence of the *fbsB* gene, which encodes another fibrinogen-binding protein, FbsB. According to the previous reports, the *fbsB* gene does not seem to participate in the attachment of S. agalactiae to fibrinogen; however, it plays a crucial role in host cell invasion by these bacteria (16). It would be interesting to complete the characteristics of isolates from our own collection in terms of the presence of the *fbsB* gene to attempt to determine whether the FbsB protein is an important factor in the pathogenesis of mastitis in cows.

The Sip surface protein is highly conserved in S. agalactiae and seems to be present in all strains. Studies have shown that this protein confers protection against GBS infections (34). The *sip* gene was detected in all isolates tested in our study.

Our findings show that the gene which determines the production of the Rib surface protein (*rib*) and the genes encoding the α (*bca*) and β (*bac*) antigens of the C surface protein were less prevalent among S. agalactiae strains. In our research, the *rib* gene was detected in 10.3% of the isolates, whereas in previous studies, this gene was present in 25.9% (27) and 89% (34) of S. agalactiae isolates derived from cases of bovine subclinical mastitis, 33% of isolates from clinical mastitis (28), and 59% of isolates from both types of mastitis (26). In this study, the *bca* gene, encoding αC protein, was found in 8.8% of the isolates, whereas other studies have reported it in 3.4% (3), 36% (26), 64.7% (24), and 78.9% (35) of S. agalactiae isolates derived from milk of cows with clinical or subclinical mastitis, and in 37% (28) and 49.3% of isolates from cows with clinical mastitis (32). On the other hand, in some studies, the *bca* gene has not been detected in bovine isolates from subclinical mastitis (27, 36). The *bac* gene, which encodes the β antigen of the C surface protein, was found in 16.2% of isolates in our study, similar to another study which reported that 12.3% of isolates derived from cows with clinical mastitis were *bac*-positive (32). No isolates with the *bac* gene were detected in some reports on S. agalactiae isolated from clinical and subclinical mastitis (3, 24, 26, 35), subclinical mastitis (36), and clinical mastitis in Polish dairy herds (28).

Previous studies have revealed that, unlike in clinical GBS strains from humans, the *scpB* gene encoding the C5a peptidase is not usually present in most tested bovine isolates (35, 36), which suggests that it is not essential for the ability of these pathogens to cause mastitis in cattle (37). Zhang et al. (32) have reported the occurrence of the *scpB* gene in 81.4% of isolates from clinical mastitis, whereas Duarte et al. (24, 35) found this gene in 50.6 and 65.8% of the tested S. agalactiae isolates from clinical or subclinical mastitis. Jain et al. (27) and Rato et al. (33) have detected the gene in 22.2 and 21.7% of isolates from bovine subclinical mastitis, respectively. Similar results were obtained in our research, in which the percentage of isolates carrying this gene was 35.3%. The *scpB* gene has not been found in some studies on bovine isolates derived from cows presenting clinical or subclinical mastitis (25, 26), or from clinical mastitis in Poland (28).

The outcome of an infection of the bovine mammary gland is dependent on the virulence of the causative pathogen and the host immunity (38, 39). In our study, no association was detected between the presence of individual virulence genes and the type of mastitis. In case of other mastitis pathogens, none of the virulence factors of Escherichia coli were associated with the persistence of intramammary infections (40), and the number of virulence factors of Staphylococcus aureus was not associated with disease severity in mastitis in cattle (41).

The MTT and LDH assays are colorimetric methods used to measure bacterial cytotoxicity caused by bacterial pathogens (25, 42). Kidney epithelial cell lines (SK, Vero) have previously been efficiently used for this type of research (42, 43). In our study, cytotoxicity assessments based on LDH and MTT assays revealed that most of the S. agalactiae isolates were noncytotoxic or exhibited a low cytotoxic effect against SK and Vero cells. However, several isolates showed a medium cytotoxic effect to these cell lines at the concentration of bacterial suspension used in this study. To the best of our knowledge, there are only few reports on the cytotoxicity of field S. agalactiae isolates recovered from bovine mastitis. For example, Pang et al. (25) studied cytotoxicity among different groups of S. agalactiae bacteria depending on their source (bovine, human, fish, and environment) and reported that all groups were cytotoxic to the examined cells in a concentration-dependent manner, with the cytotoxic effect increasing with increasing concentrations of bacterial suspension. Other reports concern the cytotoxicity of clinical mutant S. agalactiae strains with the genes encoding some virulence factors deleted (14). Both in those studies and in our own research, the LDH and MTT tests turned out to be useful for differentiating noncytotoxic isolates from those with low, moderate, and high cytotoxic effects. Moreover, a statistically significant and moderate positive correlation was found between the presence of the *scpB* virulence gene and the cytotoxicity of the isolates toward Vero cells in the MTT test.

In conclusion, our research confirms the prevalence of various virulence genes in S. agalactiae isolated from Polish dairy herds, which have previously been found in isolates from various human and animal infections. For the first time, the presence of *bac*- and *scpB*-positive isolates of S. agalactiae was confirmed in Polish dairy cattle. Moreover, the cytotoxicity of Polish bovine isolates was assessed for the first time. Some of the genes (*cspA*, *fbsA*, *hylB*, *sip*) were screened for the first time in Polish bovine isolates. Studies on the characteristics of Polish S. agalactiae isolates will enable us to update our knowledge about this pathogen. A recent report indicates that the results of molecular epidemiological studies of S. agalactiae in some regions cannot be extrapolated to other regions (44). Further studies are therefore necessary to determine the molecular epidemiology and variability of S. agalactiae isolated from Polish dairy cattle, with the aim of improving mastitis control programs with regard to S. agalactiae in this country.

## MATERIALS AND METHODS

### Bacterial isolates and sample collection.

A total of 68 S. agalactiae isolates were isolated from milk or mammary secretions from 68 Holstein-Friesian cows presenting clinical (*n* = 48) and subclinical (*n* = 20) mastitis between 2009 and 2012. The isolates came from 68 dairy farms (herds) located in different regions of Poland (13 from 16 provinces). To avoid the examination of epidemiologically related isolates, only one isolate per dairy herd was used in this study. Samples of milk or inflammatory secretions of the mammary gland were taken aseptically by field veterinarians, cooled to 4°C or frozen to −20°C, and transported to the laboratory. After the samples had reached room temperature, they were mixed and streaked (0.01 mL) on agar medium with the addition of 5% sheep blood (GRASO). The plate was incubated at 37°C in an aerobic atmosphere for 18 to 24 h. In the absence of a growth culture, the samples were incubated for an additional 24 h to allow the growth of slow-growing microorganisms. Milk samples with visible macroscopic changes were inoculated onto sugar broth to multiply the microorganisms or release them from inside the immune cells, and subsequently streaked on blood agar medium again.

The clinical condition of the udder was assessed by a veterinarian during sample collection. Milk samples (or mammary gland secretions) were evaluated in the laboratory based on somatic cell counts obtained using the Somacount 150 (Bentley Instruments, Inc.) and microbiological test results. The clinical form of mastitis was determined based on the presence of general symptoms (lack of appetite, increased body temperature), udder changes (edema, redness, soreness), macroscopic changes in mammary secretions (change in smell, consistency, presence of blood, pus, milk clots, clumps of fibrin), an increased number of somatic milk cells, and pathogenic microorganisms. The subclinical form of mastitis was diagnosed based on an increased number of somatic cells (over 200,000 cells in 1 mL), the presence of microorganisms in milk, and the absence of external signs of disease (45).

### Reference strains.

Besides field S. agalactiae strains, the following reference strains obtained from the American Type Culture Collection (ATCC) (LGC Standards) were used for quality control: Enterococcus faecalis ATCC 29212 (negative control for catalase test), Staphylococcus aureus ATCC 25923 (CAMP test, positive control for catalase test), and Streptococcus agalactiae ATCC 13813 (positive control for *cfb*, *cspA*, *fbsA*, *hylB*, *rib*, and *sip* genes). All S. agalactiae isolates and reference strains were kept at −70°C in Microbank cryovials (Biocorp).

### Identification of the isolates using phenotypic methods.

Bacteria were cultured using standard microbiological methods as described previously (29). S. agalactiae colonies were initially identified by colony morphology, type of hemolysis on agar medium with 5% sheep blood, Gram-staining (Color Gram 2 kit; bioMérieux, Marcy-l’Étoile, France), and a catalase test. The growth of catalase-negative isolates was assessed on Edwards medium with Chodkowski’s modification (29). Subsequently, S. agalactiae isolates were distinguished from other esculin-negative streptococci using a CAMP test (29). In cases of doubt, API Strep (bioMérieux) tests were additionally used.

### DNA isolation.

Under aseptic conditions, a single inoculated bead was removed from the cryovial (Microbank, Biocorp) and directly streaked onto agar with 5% sheep blood (GRASO). Plates were incubated at 37°C for 24 h, and subsequently, bacterial material derived from a single colony was streaked again to obtain a pure culture. After incubation for 24 h at 37°C, the culture was inoculated in 1 mL of brain heart infusion broth (Oxoid) and then incubated at the same temperature. A commercial Genomic Mini AX Bacteria kit (A&A Biotechnology, Gdańsk, Poland) was used to isolate genomic DNA from an 18-h bacterial culture. The DNA obtained in the final stage of isolation was suspended in 300 μL of Tris-EDTA buffer (10 mM; Promega, Madison, WI) and stored at −20°C.

### Detection of the genes with PCR.

Phenotypic identification of S. agalactiae isolates was confirmed by standard PCR. Subsequently, virulence factor genes of S. agalactiae (*bac*, *bca*, *cfb*, *cspA*, *cylE*, *fbsA*, *hylB*, *rib*, *scpB*, and *sip*) were tested for all isolates. The concentrations of the components included in the reaction mixtures used for the amplification of gene fragments were selected experimentally and based on the source literature. The reaction mixture, at a final volume of 20 μL, contained 0.1 μM of each primer (0.4 μM for *bca* and *rib*), 0.2 mM each deoxynucleotide triphosphate, 2 to 5 mM MgCl_2_, Green GoTaq Flexi Buffer, 0.5 U of GoTaq Flexi DNA polymerase (0.25 U for *bca* and *rib*), and 1 μL of genomic DNA. All reagents were obtained from Promega (Madison, WI). The primer sequences and conditions used for amplification of DNA fragments are presented in Table 4. The reactions were carried out in a TProfessional Thermocycler (Biometra Ltd.) or a C1000 Touch Thermal Cycler (Bio-Rad, Hercules, CA). A positive control (DNA isolate containing the tested gene) and a negative control (nuclease-free water) were included with each reaction.

**TABLE 4:** PCR primers and cycling conditions used to identify and characterize Streptococcus agalactiae isolates (*n* = 68) from milk of dairy cows with clinical or subclinical mastitis

Gene	Primer sequence (5′→3′)	Amplicon size (bp)^*a*^	MgCl_2_ concn (mM)	Reference
16S−23S rRNA	Fw: TGTTTAGTTTTGAGAGGTCTTG	150^*b*^	3	47
	Rv: CGTGGAATTTGATATAGATATTC			
16S−23S rRNA	Fw: GGAAACCTGCCATTTGCG	281^*b*^	5	47
	Rv: TAACTTAACCTTATTAACCTAG			
*bac*	Fw: AAGCAACTAGAAGAGGAAGC	479^*c*^	2	48
	Rv: TTCTGCTCTGGTGTTTTAGG			
*bca*	Fw: TGATACTTCACAGACGAAACAACG	398^*d*^	2	49
	Rv: TACATGTGGTAGTCCATCTTCACC			
*cfb*	Fw: TTTCACCAGCTGTATTAGAAGTA	153^*e*^	3	30
	Rv: GTTCCCTGAACATTATCTTTGAT			
*cspA*	Fw: CGAAGTTCCTGGTTCAGAAGATT	574^*f*^	5	50
	Rv: TACTGCAGGACGAGCTTTGAAG			
*cylE*	Fw: TGACATTTACAAGTGACGAAG	268^*g*^	5	51
	Rv: TTGCCAGGAGGAGAATAGGA			
*fbsA*	Fw: GTAGGTCAACTTATAGGG	289^*h*^	3	52
	Rv: ATACTTAATTTTCATTGCG			
*hylB*	Fw: CACCAATCCCCACTCTACTA	444^*c*^	5	48
	Rv: TGTGTCAAACCATCTATCAG			
*rib*	Fw: TGATACTTCACAGACGAAACAACG	295^*d*^	2	49
	Rv: CATACTGAGCTTTTAAATCAGGTGA			
*scpB*	Fw: CCTGCTAAGACTGCTGATAC	853^*c*^	5	48
	Rv: CATAAGCATAGTCGTAAGCC			
*sip*	Fw: TGAAAATGCAGGGCTCCAACCTCA	293^*i*^	5	53
	Rv: GATCTGGCATTGCATTCCAAGTAT			

a PCR temperatures and conditions are shown in the table footnotes.

b 94°C (600 s); 30 cycles of 94°C (60 s), 55°C (60 s), 72°C (60 s); final extension 72°C (420 s).

c 94°C (300 s); 30 cycles of 94°C (30 s), 53°C (30 s), 72°C (60 s); final extension 72°C (240 s).

d 96°C (180 s); 30 cycles of 95°C (60 s), 58°C (45 s), 72°C (45 s); final extension 72°C (600 s).

e 94°C (180 s); 40 cycles of 95°C (1 s), 55°C (30 s), 72°C (120 s); final extension 72°C (300 s).

f 94°C (600 s); 35 cycles of 95°C (60 s), 53°C (30 s), 60°C (120 s).

g 95°C (600 s); 35 cycles of 95°C (60 s), 55°C (60 s), 72°C (120 s); final extension 72°C (420 s).

h 95°C (30 s); 44 cycles of 95°C (1 s), 50°C (15 s), 72°C (30 s).

i 96°C (300 s); 30 cycles of 96°C (60 s), 55°C (60 s), 72°C (120 s); final extension 72°C (480 s).

### Electrophoresis.

Electrophoretic separation of the amplification products (6 μL) was carried out in agarose gel (1.5%; Analytical Grade LE Agarose; Promega) containing ethidium bromide at a concentration of 0.5 μg mL^−1^ of gel (Promega), using 1× Tris-borate-EDTA (TBE) buffer (Sigma-Aldrich) and a voltage of 10 V · cm^−1^ of gel. The separated PCR products were visualized using the ImageMaster VDS Gel Documentation System (Amersham Pharmacia Biotech, Amersham, United Kingdom). Amplicon sizes were compared with DNA markers (GeneRuler 100-bp Plus DNA Ladder; Thermo Fisher Scientific, Waltham, MA) and the PCR product was obtained by amplifying the DNA of the reference strain or isolate used as a positive control for the reaction. A positive PCR result was found when an amplicon of a certain size, expressed in bp, was obtained.

### Cytotoxicity.

Thirty-four randomly selected S. agalactiae isolates examined in the study were subjected to cytotoxicity testing using two methods: the LDH and the MTT tests.

The cytotoxicity of S. agalactiae isolates toward a SK cell line (swine kidney epithelial cells) and a Vero cell line (African green monkey kidney epithelial cells) was determined using the *in vitro* cell-based method with lactate dehydrogenase, applying the Pierce LDH Cytotoxicity Assay kit (Thermo Fisher Scientific) according to the manufacturer’s instructions. The SK and Vero cell lines used in the experiment were derived from a cell culture collection of the Ludwig-Maximilian University of Munich and were obtained from Manfred Gareis.

The SK and the Vero cells were grown in minimum essential Eagle medium with Earle’s salts (MEM; Sigma-Aldrich, St. Louis, MO), supplemented with 5% fetal bovine serum (FBS; Sigma-Aldrich). Cells were seeded into 96-well plates 1 day before testing (1.0 × 10^4^/well) and incubated for 24 h at 37°C, 5% CO_2_, to obtain confluent growth. According to the manufacturer’s manual, the optimal number of cells/well plated in 100 μL of medium was determined in preliminary experiments. Bacterial cells were grown in brain heart infusion broth (Oxoid) at 30°C and harvested after 6 h, when it was anticipated that the culture had reached a cell density of at least 2 × 10^8^ CFU/mL (0.67 McFarland turbidity determined with a DEN-1B Densitometer, Grant Instruments, Poland). The bacterial cells were removed by centrifugation at room temperature (46). The SK and Vero cells were infected with 10 μL of bacterial culture supernatant. Additionally, MEM medium supplemented with 5% fetal calf serum (FCS) with water-treated SK/Vero cells was included as a spontaneous control, and SK/Vero cells treated (after 17 h of incubation) with lysis buffer were used as a positive control (maximum LDH activity). Additionally, serum-free MEM medium and MEM medium supplemented with 5% FCS were used to determine LDH background activity; absorbance was measured at 492 and 690 nm, and cytotoxicity was calculated according to the formula included in the manufacturer’s manual.

The tested bacterial isolates were considered cytotoxic if the calculated values were above 20% of the absorbance obtained from the maximum LDH activity (the SK and the Vero cells treated with 10× lysis buffer) (46). Therefore, the cytotoxicity test results were defined as either ‘cytotoxic’ or ‘noncytotoxic.’

The MTT assay was performed as described by El-Housseiny et al. (42), with minor modifications. The Vero and SK cells were maintained in TPP cell culture flasks (Techno Plastic Products AG, Switzerland) containing minimum essential Eagle medium with Earle’s salts (Sigma-Aldrich) supplemented with 2% fetal bovine serum (FBS; Sigma-Aldrich) and 100 μg mL^−1^ streptomycin at 37°C in a humidified atmosphere of 5% CO_2_ and 95% air. Prior to conducting the experiment, S. agalactiae isolates were cultured in trypticase soy broth supplemented with 5% defibrinated sheep blood. Each S. agalactiae isolate was added to one set of triplicate wells containing cells. The cytotoxicity measurement was carried out at 510 nm, and cytotoxicity was defined as low (<50%), moderate (50 to 85%), or high (>85%) (42).

### Statistical analyses.

Statistical analysis was performed with MS Excel 365 and Statistica version 13.3 (Microsoft 2020, Statsoft 2019). A chi-squared test, contingent coefficient, and Spearman’s correlation were used to assess the association between the presence of individual virulence genes and the type of mastitis. *P ≤ *0.05 was considered to be significant and *P ≤ *0.001 was considered to be highly significant.

A chi-squared test with Yates continuity correction and Spearman’s correlation was used to determine the correlation of the presence of virulence genes with cytotoxicity toward the SK and Vero cell lines.
