# Supplementary material for: Virulence Factor Genes and Cytotoxicity of Streptococcus agalactiae Isolated from Bovine Mastitis in Poland
Source: Microbiol Spectr. 2022 May 24;10(3):e02224-21. doi: 10.1128/spectrum.02224-21 (PMC9241884; doi:10.1128/spectrum.02224-21)
Supplement: SUPPLEMENTAL FILE 1 — Supplemental material. Download spectrum.02224-21-s001.pdf, PDF file, 0.5 MB [file spectrum.02224-21-s001.pdf]

Supporting Information

TABLE S1 Results of the statistical analysis used to determine differences in the prevalence of virulence genes among *Streptococcus agalactiae* isolates (n=68) derived from clinical and subclinical cases of mastitis

**Statistical parameters**		**Virulence genes**					
		bac	***bca***	***cylE***	***fbsA***	***rib***	***scpB***
Chi-squared test and Contingent coefficient	X^2	2.61	0.52	0.25	0.29	0.01	0.28
	p	0.1062	0.4730	0.6202	0.5893	0.9589	0.6002
	ϕ	0.201	0.087	0.137	0.065	0.02	0.06
Spearman's correlation	R_{Spr}	0.20	0.09	0.14	0.07	0.02	0.07
	p	0.1093	0.4805	0.2594	0.5958	0.9597	0.6066
U-Manna Whitney test	U	404.0	454.0	450.0	462.0	478.0	448.0
	p	0.3095	0.7314	0.6913	0.8138	0.9839	0.6716

$p \leq 0.05$ or ≤ 0.001 was considered statistically significant

TABLE S2 Results of the statistical analysis[a] used to determine a correlation of the presence of *Streptococcus agalactiae* virulence genes with a cytotoxicity of the *S. agalactiae* isolates towards the SK and the Vero cell lines according to the MTT test

**Statistical parameters**	**Virulence genes**											
	bac		***bca***		***cylE***		***fbsA***		***rib***		***scpB***	
	**Cell line**											
	**SK**	**VERO**	**SK**	**VERO**	**SK**	**VERO**	**SK**	**VERO**	**SK**	**VERO**	**SK**	**VERO**
X^2_{Yates}<br>*p*	0.57;<br>0.451	0.17;<br>0.681	0.03;<br>0.868	1.03;<br>0.598	0.36;<br>0.549	0.01,<br>0.959	0.12;<br>0.733	0.31;<br>0.579	0.36;<br>0.549	0.01;<br>0.960	0.01;<br>0.923	6.94;<br>0.009
R_{Spr}<br>*p*	0.22;<br>0.227	0.06;<br>0.754	-0.08;<br>0.670	-0.13;<br>0.471	0.23;<br>0.194	0.22;<br>0.202	-0.15;<br>0.397	0.02;<br>0.908	-0.23;<br>0.194	-0.22;<br>0.202	-0,05;<br>0.805	0.42;<br>0.013

[a] Statistical analysis was performed only for those virulence factors that were not present in all *Streptococcus agalactiae* isolates

$p \leq 0.05$ or ≤ 0.001 was considered statistically significant

TABLE S3 Results of the statistical analysis[a] used to determine a correlation of the presence of *Streptococcus agalactiae* virulence genes with a cytotoxicity of the *S. agalactiae* isolates towards the SK and the Vero cell lines according to the LDH test

**Statistical parameters**	**Virulence genes**											
	bac		***bca***		***cylE***		***fbsA***		***rib***		***scpB***	
	**Cell line**											
	**SK**	**VERO**	**SK**	**VERO**	**SK**	**VERO**	**SK**	**VERO**	**SK**	**VERO**	**SK**	**VERO**
X^2_{Yates}	-[b]	0.08;	-	0.05;	-	0.82,	-	0.62;	-	0.82;	-	1.39;
p	-	0.774	-	0.829	-	0.365	-	0.431	-	0.365	-	0.239
R_{Spr}	-	0.03;	-	-0.07;	-	-0.28;	-	-0.23;	-	-0.28;	-	0.26;
p	-	0.847	-	0.704	-	0.107	-	0.196	-	0.107	-	0.135

[a] Statistical analysis was performed only for those virulence factors that were not present in all *Streptococcus agalactiae* isolates

[b] Due to the lack of variability for the cytotoxicity, analysis could not be performed for the SK cell line in the LDH test

$p \leq 0.05$ or ≤ 0.001 was considered statistically significant
